# Blooms of Toxic Cyanobacterium *Nodularia spumigena* in Norwegian Fjords During Holocene Warm Periods

**DOI:** 10.3390/toxins12040257

**Published:** 2020-04-15

**Authors:** Robert Konkel, Anna Toruńska-Sitarz, Marta Cegłowska, Žilvinas Ežerinskis, Justina Šapolaitė, Jonas Mažeika, Hanna Mazur-Marzec

**Affiliations:** 1 University of Gdańsk, Faculty of Oceanography and Geography, Division of Marine Biotechnology, Marszałka J. Piłsudskiego 46, PL-81-378 Gdynia, Poland; robert.konkel@phdstud.ug.edu.pl (R.K.); anna.torunska@ug.edu.pl (A.T.-S.); 2 Institute of Oceanology, Polish Academy of Science, Powstańców Warszawy 55, PL-81-712 Sopot, Poland; mceglowska@iopan.pl; 3 Mass Spectrometry Laboratory, Center for Physical Sciences and Technology, LT-10257 Vilnius, Lithuania; zilvinas.ezerinskis@ftmc.lt (Ž.E.); justina.sapolaite@ftmc.lt (J.Š.); 4 Laboratory of nuclear geophysics and radioecology, Nature research Centre Akademijos Str. 2, LT-08412 Vilnius, Lithuania; jonas.mazeika@gamtc.lt

**Keywords:** nodularin, anabaenopeptins, genetic markers, Holocene climate optimum, sediment cores, Norwegian fjords

## Abstract

In paleoecological studies, molecular markers are being used increasingly often to reconstruct community structures, environmental conditions and ecosystem changes. In this work, nodularin, anabaenopeptins and selected DNA sequences were applied as *Nodularia spumigena* markers to reconstruct the history of the cyanobacterium in the Norwegian fjords. For the purpose of this study, three sediment cores collected in Oslofjorden, Trondheimsfjorden and Balsfjorden were analyzed. The lack of nodularin in most recent sediments is consistent with the fact that only one report on the sporadic occurrence and low amounts of the cyanobacterium in Norwegian Fjords in 1976 has been published. However, analyses of species-specific chemical markers in deep sediments showed that thousands of years ago, *N. spumigena* constituted an important component of the phytoplankton community. The content of the markers in the cores indicated that the biomass of the cyanobacterium increased during the warmer Holocene periods. The analyses of genetic markers were less conclusive; they showed the occurrence of microcystin/nodularin producing cyanobacteria of Nostocales order, but they did not allow for the identification of the organisms at a species level.

## 1. Introduction

In Norwegian coastal waters, the phytoplankton community is mainly composed of diatoms, dinoflagellates, cyanobacteria, prymnesiophytes and raphidophytes [1,2,3]. Some of the organisms produce acute toxins and/or form harmful blooms (HABs). Their occurrence, even in low amounts, can result in the closure of shellfish harvesting areas. The studies performed by Lännergren [4] in Lindaspollene, a land-locked fjord system on the west coast of Norway (north of Bergen), revealed the sporadic occurrence of heterocystous *Nodularia* sp. This cyanobacterium was found there in small amounts, and no data on its toxicity was reported. In many brackish water bodies, *N. spumigena* regularly develops into toxic blooms, threatening public health and decreasing the recreational value of bathing sites [5]. This planktonic cyanobacterium, among others, was reported in the Baltic Sea [6], estuaries, rivers and lakes in Australia [7], New Zealand [8], the North Sea [9] and the Black Sea [10]. However, no records on its massive occurrence in the Norwegian coastal waters are available. The species produces a wide array of bioactive nonribosomal peptides, including the potent hepatotoxic nodularin (NOD), anabaenopeptins (APs), spumigins (SPUs) and aeruginosins (AERs) [11,12]. Analyses of NOD, APs and species-specific gene sequences in deep sediment samples made it possible to reconstruct the history of the species in the Baltic Sea [13]. The study proved, for the first time, the thousands-year presence of *N. spumigena* in the sea. The successful application of molecular (chemical and genetic) markers in that study encouraged us to use the same tools in the reconstruction of toxic algal blooms in Norwegian fjords. The chemical analysis of deep sediment cores performed with the application of LC-MS/MS did not reveal the presence of saxitoxin (STX), okadaic acid (OA) or dinophysiotoxin DTX-1 (data not published). Unexpectedly, in some sediment samples, NOD and APs were detected, indicating that in the past, *N. spumigena* was present in Norwegian fjords.

The aim of the present study was to gain knowledge about the occurrence and intensity of *N. spumigena* blooms in Norwegian fjords. For the purpose of the study, we assumed that the presence and content of NOD in sediments would roughly correspond to the presence and intensity of *N. spumigena* blooms. A correlation between biomass indicators (i.e., chlorophyll a [14] and the remotely determined turbidity index [15]), and NOD concentration in *N. spumigena* bloom samples was previously documented. The selection of NOD as a proxy indicator of *N. spumigena* biomass was additionally justified by the fact that *N. spumigena* is the main and almost the only NOD-producing planktonic cyanobacterial species. The rare examples of the production of this toxin by other cyanobacteria include *Nostoc* from benthic habitats of the saline-alkaline lake in Brazil [16], lichen thalli from Kenya and Argentina [17], cycads [*Nostoc* spp. ‘*Macrozamia riedlei* 65.1’ and ‘*Macrozamia serpentina* 73.1’, [18], and *Iningainema pulvinus* from Australian freshwater spring wetland [19]. To demonstrate that *N. spumigena* was a source of NOD in deep sediments from the Norwegian coastal waters, in our work, the anabaenopeptin variants specific for *N. spumigena* and selected DNA sequences were also analyzed.

## 2. Results

### 2.1. N. spumigena Chemical Markers in Sediment Samples

**Figure 1:**
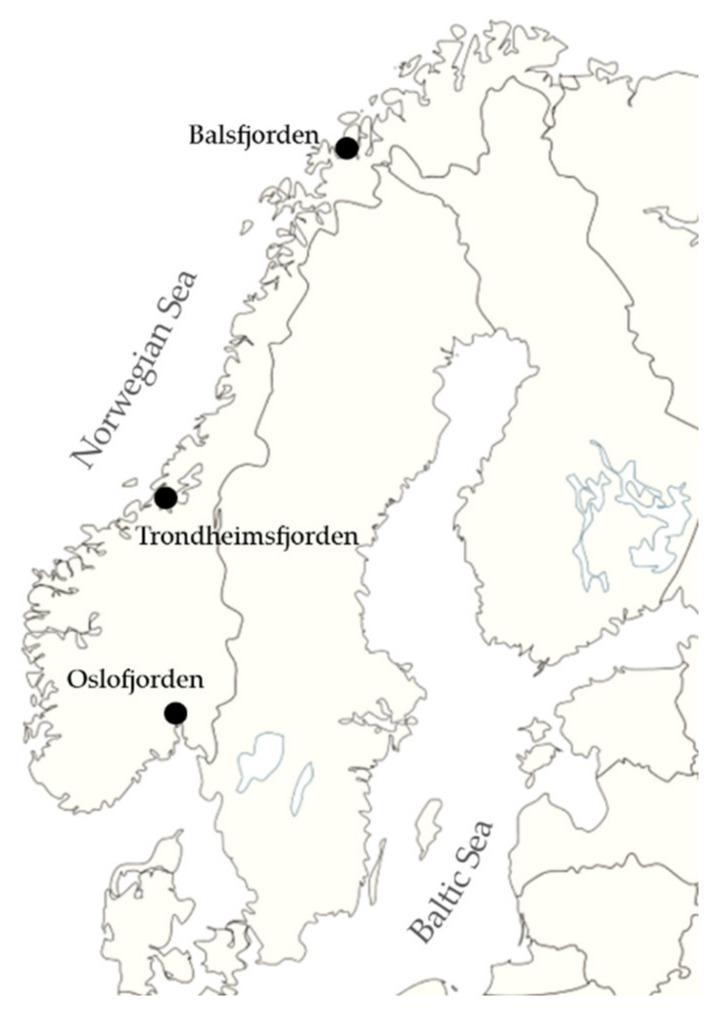
Location of the sampling stations in the Norwegian coastal waters.

In the study, we analyzed sediment samples from the short (SC) and long (LC) cores collected in Norwegian fjords, namely in Oslofjorden (336 cm), Trondheimsfjorden (136 cm) and Balsfjorden (440 cm) (Figure 1).

**Figure 2:**
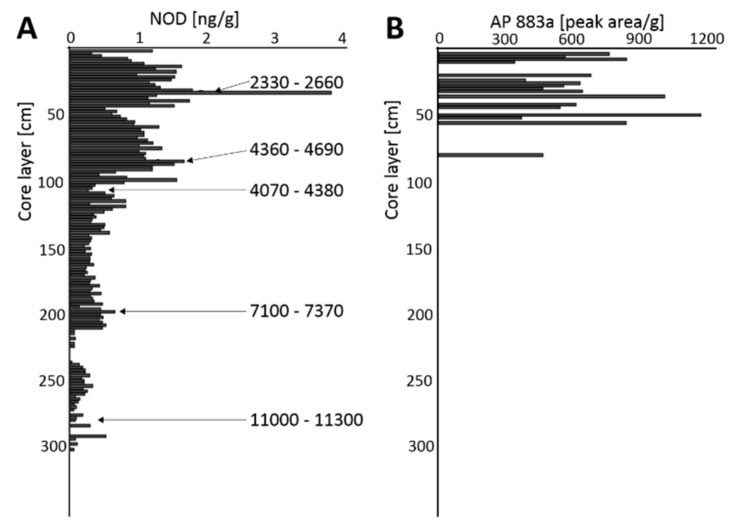
Nodularin (NOD) (**A**) and anabaenopeptin AP883a (**B**) contents in the long sediment core (LC) from Oslofjorden. The numbers indicate the calibrated age of the sediments [cal yr BP].

The detection and identification of NOD in sediments were based on the MRM chromatograms (Figure S1) and on the mass fragmentation spectra (Figure S2). With the exception of one sample, i.e., the 8–10 cm layer of SC from Oslofjorden, no NOD was detected in the more recent sediments (Figure 2; Table S1). In case of the sediments from Oslofjorden, an increase in the NOD content was observed with depth, i.e., from the 0 to 2 cm layer of the LC, up to the maximum value, 3.84 ng/g dw, in the 32–34 cm layer (Figure 2; Table S1). According to the dating (Table S2, Figure S3), these sediments were deposited 2330–2660 cal yr BP (calibrated years before present) (Figure 2, Table S1). After a decline in the 44–46 cm layer (0.52 ng/g), the NOD content started to increase again, and in the 84–86 cm layers, dated to 4360–4690 cal yr BP, reached 1.68 ng/g. Then, the content of NOD dropped, and in the 102–212 cm layers, stayed at a stable level of 0.14–0.82 ng/g (Figure 2; Table S1). In case of the deeper part of the core from Oslofjorden (212–304 cm), either negative results (i.e., no signal or S/N < 3) were obtained (14 samples) or the NOD content was low (0.03–0.53 ng/g). No NOD was found below 304 cm. The sediments deposited 11,000–11,300 cal yr BP (278–280 cm layer) and some of the older sediments, still contained detectable amounts of the toxin. In the samples from Oslofjorden, anabaenopeptin AP883a was also found. The peptide was detected in one sample from SC (8–10 cm) (Table S1) and in 19 of the 29 samples from the upper section of the LC (0–58 cm), where the NOD content was the highest (Figure 2; Table S1). As for the deeper sediments (>58 cm), AP883a was found only in 3 layers of the core.

**Figure 3:**
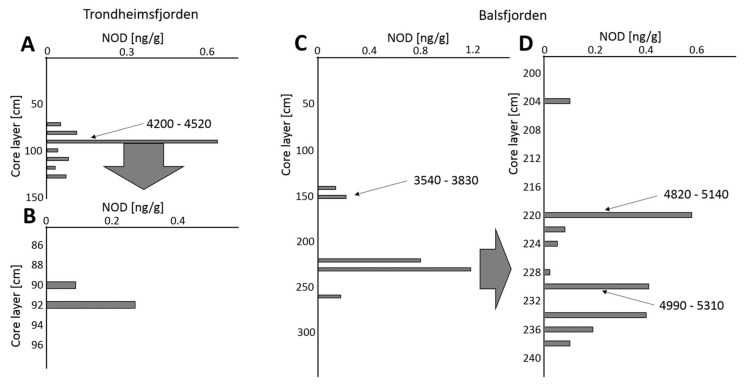
Nodularin (NOD) content in the integrated 10-cm sections (**A**) and 2 cm layers (**B**) of the long sediment core (LC) from Trondheimsfjorden and in the integrated 10 cm sections (**C**) and 2 cm layers (**D**) of the long sediment core from Balsfjorden. The numbers indicate the calibrated age of the sediments [cal yr BP].

The integrated sediment samples from Trondheimsfjorden did not reveal the presence of NOD in the upper 0–60 cm section of the LC. However, NOD was found in all deeper 10 cm samples, with a maximum in the 90–100 cm layer (0.63 ng NOD/g) (Figure 3A; Table S3). The analysis of 2 cm samples of this section showed the presence of NOD only in the 90–92 cm (0.09 ng/g) and 92–94 cm layers (0.27 ng/g) dated to 4200–4520 cal yr BP. In these two sediment layers, AP883a was also present (Table S3).

In the LC from Balsfjorden, NOD was not found in the top 140 cm section. The toxin was detected in the following integrated 10 cm sections of the core: 140–150 cm (0.14 ng/g), 150–160 cm (0.22 ng/g), 220–230 (0.80 ng/g), 230–240 (1.19 ng/g) and 260–270 cm (0.18 ng/g) (Figure 3, Table S4). As for the 2 cm samples, the toxin was present in trace amounts (S/N < 5) in the 152–154 cm layer deposited 3540–3830 cal yr BP, and in nine layers from Section 204–240 cm (0.02–0.58 ng NOD/g) (Figure 3, Table S4). According to radiocarbon dating (Table S2), the sediments from the layers 220–222 cm (0.58 ng NOD/g) and 230–232 cm (0.41 ng NOD/g) were deposited 4820–5140 cal yr BP and 4990–5310 cal yr BP, respectively (Figure 3, Table S4). As in the cores from Oslofjorden and Trondheimsfjorden, the sediments from Balsfjorden also contained anabaenopeptin AP883a. This peptide marker was found only in the 220–222 cm layer (AP883a) where the NOD content was the highest (Table S4). In addition, the integrated 150–160 cm sample contained another anabaenopeptin variant, AP827.

### 2.2. Genetic Markers in Sediment Samples

DNA extracted from SC and LC sediments collected at the three Norwegian stations differed in terms of quality and quantity, as shown in Table 1. The quantities of DNA obtained from SC sediments from Oslofjorden, especially from its surface layer, were higher (100–242 ng/μL) than those from the deepest parts of the LC (31–50 ng/μL). DNA was also successfully extracted from all three sediment samples from Balsfjorden and the 92–94 cm layer from Trondheimsfjorden, but we failed to isolate DNA from the deepest sediment layer (134–136 cm) from Trondheimsfjorden (Table 1).

In case of SC, only the cyanobacterial 16S rDNA sequences were detected, and only in the samples from Oslofjorden (Table 1). As for the LCs, the PCR gave products indicating the presence of cyanobacterial 16S rDNA fragments in samples from all stations, including the deepest layers of the Oslofjorden and Balsfjorden sediments (Table 1). The cyanobacterial *cpcBA*-IGS sequences were detected in seventeen layers of the LC from Oslofjorden, from the 32–34 cm to the 338–340 cm layer, and also in 92–94 cm layer of the core from Trondheimsfjorden. The amplification of *mcyE*/*ndaF* gene gave PCR products only in thirteen LC sediment samples from Oslofjorden, from the 34–36 cm to the 362–364 cm layer.

The sequencing of the PCR products was successful only in the cases of 16S rDNA fragments derived from the 84–86 cm layer (565 bp) deposited 4360–4690 cal yr BP, and from the 148–150 cm (650 bp) and 232–234 cm (549 bp) layers of the LC collected in Oslofjorden. These sequences were 100% similar and showed 99–100% similarity to the sequences of the cyanobacteria from *Nostocales* order, namely *Aphanizomenon*, *Dolichospermum* and *Anabaena*, deposited in GenBank (Figure S4). They were also more than 93% similar to sequences from *Nostoc* sp. and three *N. spumigena* genomes (retrieved from NCBI).

## 3. Discussion

*N. spumigena* is a brackish water species; its growth is favored by eutrophication and increased water temperature [6]. Due to its N_2_-fixation capability [6], as well as its utilization of organic phosphorous [20], the cyanobacterium can grow even under limited availability of dissolved inorganic nutrients. The mass development of the cyanobacterium in the Baltic Sea during warm summers confirmed the effect of temperature on *N. spumigena* growth. The determined NOD content in the Baltic sediments documented the current state and the history of the toxic *N. spumigena* blooms in this ecosystem. In the surface (0–5 cm), i.e., the most recent sediments, the average content of NOD was 3.9 ng/g [21]. In the 0–14 cm layers of the sediment core collected from the Gulf of Gdańsk, NOD ranged from 1.05 to 4.32 ng/g [13]. An extremely high level of nodularin (225.94 ng NOD/g) was detected in the deeper parts of the same core [13]. These sediments were deposited during Roman Climate Optimum (1600–2700 BP) [22] when the average summer temperature was only slightly lower than in the late 20th century [23]. Based on this finding, it was concluded that in the past, the blooms of the toxic cyanobacterium in the Baltic Sea were even more intense than today.

As for the Norwegian coastal waters, data on the presence of cyanobacteria are limited, and mainly refer to picocyanobacteria of the genus *Synechococcus* [24,25]. In Lindaspollene, a land-locked fjord system north of Bergen, the nitrogen-fixing cyanobacteria *Calothrix confervicola*, *Anabaena torulosa* and *Rivularia* sp. were observed [4]. Low amounts and sporadic occurrence of *Nodularia* sp. were reported only once [4]. This fact, as well as the lack of NOD in the surface sediments analyzed in our work, indicate that nowadays in Norwegian fjords, *N. spumigena*, if present, does not constitute a noticeable component of the cyanobacterial community. Unexpectedly, NOD and *N. spumigena*-specific APs variants were found in deeper parts of the sediment cores, indicating that in the past, the toxic cyanobacterium regularly formed blooms in the fjords. Based on the level of the toxin, we assumed that the abundance of the cyanobacterium was lower than in the Baltic Sea. In LCs from the three sampling stations, the maximum NOD contents were 3.84 ng/g (Oslofjorden; 32–34 cm), 0.27 ng/g (Trondheimsfjorden; 92–94 cm) and 0.58 ng/g (Balsfjorden; 220–222 cm). NOD content in the cores seemed to be related to the reconstructed climate changes in Northern Europe. Since the early Holocene (approximately 12,000 BP), the temperature started to increase rapidly and reached a stable level in approximately 6500 BP [26]. The rise in temperature could be one of the factors that stimulated the growth of *N. spumigena* in Norwegian fjords. As we discovered, in Oslofjorden, the NOD-producing cyanobacteria were already present over 11000 BP. Then, an increase in the NOD content was observed in sediments deposited approximately 7100–7370 cal yr BP, when the average Holocene summer temperature reached its maximum [27,28]. It is also noteworthy that in both the Norwegian fjords and the Baltic Sea, peaks in NOD content (Table 2) coincided with another Holocene warm period which occurred over 4000–5000 PB [27]. Moreover, NOD and AP883a measurements indicated that in Oslofjorden and in the Gulf of Gdańsk, the more intense blooms of *N. spumigena* occurred approximately 2330–2660 cal yr BP during the Roman Climate Optimum (Table 2).

This period was characterized by average summer temperatures comparable to those of late 20th century summers [30]. The alternating years of slightly warmer and colder Holocene summers can explain the observed variability in NOD content in sediment layers. As shown, lower temperatures do not exclude the presence of *N. spumigena*. The cyanobacterium has been previously reported in the Arctic [31] and Antarctic regions [32], as well as in the Hopar glacier in Pakistan [33]. Chrismas et al. [34] revealed that *Nodularia* had cold-tolerant ancestors and preserved the capacity to adjust to low water temperatures. The development of brackish water *N. spumigena* in Norwegian fjords could also be supported by other factors, such as changes in water salinity caused by melting ice-sheets in the Early Holocene (9000–12,000 BP).

To strengthen the validity of the assumption that the NOD detected in the analyzed sediments derived from *N. spumigena*, additional chemical and genetic markers were applied. In recent years, ancient sedimentary DNA has been more commonly used for the reconstruction of the cyanobacterial history of both marine and lacustrine environments [13,35,36,37]. However, in our studies, the analyses of the three genetic markers, 16S rDNA, *cpc*BA-IGS and *mcyE*/*ndaF*, did not lead to conclusive results. In the case of the sediments collected in Balsfjorden and Trondheimsfjorden, only the presence of cyanobacteria was confirmed. The poor quality of the DNA isolated at these stations, weak amplification and unsuccessful sequencing were probably caused by the application of freeze-dried materials, instead of wet biomass. In the majority of other studies on ancient cyanobacterial sedimentary DNA, wet samples stored at −80 °C were used (e.g., [13,35,36,37]). However, successful analyses of cyanobacterial DNA retrieved from lyophilized sediments were also performed [38]. Nucleic acids are susceptible to oxidation, hydrolysis and bacterial degradation, and therefore, are not well-preserved in sediments. As a consequence, the analysis of sedimentary DNA represents a challenge for the researchers [39]. More detailed information was obtained from the analysis of wet sediments from Oslofjorden. The sequencing of the 16S rDNA fragment (~600 bp) indicated the presence of cyanobacteria of the Nostocales order. However, the fragment was too short to attribute it to a specific cyanobacterial genus. Moreover, the detection of *mcyE*/*ndaF* genes [40] in the sediments from Oslofjorden clearly indicated the presence of microcystin/nodularin producing cyanobacteria.

As an additional group of species-specific markers, the selected cyclic anabaenopeptins (APs) were analyzed. In *N. spumigena*, at least 36 variants of the peptides were identified [12]. A unique feature of APs produced by *N. spumigena* is the frequent occurrence of Met or Ser in a cyclic part of the molecule. To distinguish these compounds from APs produced by other cyanobacteria, they were called nodulapeptins. The two anabaenopeptins detected in sediments from the Norwegian fjords, AP883a (Ile+CO[Lys+Met+Hph+MeHph+Met]) and AP827 (Phe+CO+[Lys+Val+Hty+MeAla+Phe]) were previously reported from the Baltic *N. spumigena* strains [12]. What is more, AP883a was found to be unique to subpopulation A, while AP827 was produced only by *N. spumigena* strains classified as subpopulation B [12]. These peptide markers were also detected in deep sediment samples from the Baltic Sea [13]. Therefore, we can speculate that the *N. spumigena* subpopulations which occurred in the Norwegian fjords thousands of years ago could represent the same chemotypes as the Baltic subpopulations. The two populations probably had a common ancestor which was transported between the ecosystems through the Danish Straits. However, the available data are insufficient to indicate the direction of *N. spumigena* transfer through the straits. The analysis of deeper cores from the Baltic Sea and the Norwegian fjords could shed more light on the origin and the history of the toxic cyanobacterium in the studied ecosystems.

## 4. Conclusions

The cyanobacterial cyclic peptides, nodularin and anabaenopeptins were confirmed to be specific and stable markers which are useful in the study of the history of toxic *N. spumigena* blooms.

In previous studies, we proved that in the past, during Roman Climate Optimum, *N. spumigena* blooms in the Baltic Sea were more intense than today. In the current work, we showed that although today this cyanobacterium is not recorded in the phytoplankton community of Norwegian fjords, its blooms were common there in the warm Holocene periods. The results of this study confirmed the significance of temperature on *N. spumigena* development. They also indicated that the climate changes observed today may result in a wider spread and more massive blooms of the cyanobacterium.

## 5. Materials and Methods

### 5.1. Sediment Sampling

The sampling was performed at three stations located in Norwegian coastal waters: Oslofjorden, Trondheimsfjorden and Balsfjorden (Figure 1, Table 3). The upper sediment layers were collected with a gravity corer (short core), while for the collection of the long cores, a vibrocorer was used. The sediment cores were sliced into 2 cm-thick layers. In case of the Oslofjorden, the core was divided into two subsamples: those assigned for chemical analysis were lyophilized and those for genetic analysis were immediately frozen and stored at −80 °C. In case of the material from Trondheimsfjorden and Balsfjorden, only the lyophilized sediment samples were available for our work.

### 5.2. Extraction and LC-MS/MS Analysis

The extraction was performed following the procedure described by Cegłowska et al. [13]. In order to obtain a homogenous sample, the lyophilized material was first ground in a mortar. Then, approximately 2 g of the 2-cm sediment layers of the core from Oslofjorden and the selected 2 cm layers of the cores from Balsfjorden and Trondheimsfjorden were extracted in 5 mL of 75% methanol in water by vortexing (10 min) and bath sonication (15 min). The samples were centrifuged (12,000 g; 15 min; 8 °C) and the obtained supernatants were transferred to another tube. The procedure was repeated with a 2.5 mL portion of the same solvent. The combined extracts were evaporated to dryness in a centrifugal vacuum concentrator (MiVac, UK). In the case of sediments from Balsfjorden and Trondheimsfjorden, the integrated 10 cm layers of sediments were analyzed first to identify the sections in which NOD was present. In these analyses, five 2 g samples of each 2 cm layer from the 10 cm sections of the cores were thoroughly mixed and extracted with 30 mL of solvent (20 mL + 10 mL). Before the LC-MS/MS analysis, the samples were dissolved in 1 mL of 75% methanol. The sediment extracts were screened for the presence of *N. spumigena* chemical markers, namely NOD and *N. spumigena* specific anabaenopeptins. For these analyses, an Agilent 1200 HPLC system (Agilent Technologies, Waldbronn, Germany) linked to a hybrid triple quadrupole/linear ion trap mass spectrometer (QTRAP5500, Applied Biosystems, Sciex, Concorde, ON, Canada) was used [13]. In multiple monitoring mode (MRM), the following transitions (→) were monitored: 825 → 135 (quantifier, collision energy CE 80; LOQ = 0.05 ng/g dw; S/N > 5), 825 → 227 (CE 65), and 825 → 163 (CE 60) for NOD; 828 → 637, 405, 120, and 84 (at CE 60) for AP827; and 884 → 689, 511, 339, 164 and 107 (at CE 60) for AP883a. The results were considered to be significant when at the retention time characteristic for the standard NOD (9.63 min) a peak with the same transitions as NOD and the S/N > 5 occurred. In addition, to confirm the presence of the peptide detected in MRM mode, the fragment ion mass spectra were collected in enhanced product ion mode.

### 5.3. Genetic Analysis

For DNA analysis, the sediment samples (200 mg lyophilized material or 500 mg wet biomass) listed in Table 1 were used. The DNA extractions were performed using FastDNA™ Kit for Soil (MP Biomedicals, Santa Ana, CA, USA). The quality and quantity of the isolated DNA were determined as described in Cegłowska et al. [13]. All polymerase chain reactions were performed in a Mastercycler^®^ nexus GSX1 (Eppendorf, Hamburg, Germany). For amplification of the 16S rDNA gene, the combinations of two primers 27F and 809R [41], as well as 359F and 23S30R [32,42], were used. Amplification of the *cpcBA*-IGS with PCβF and PCαR primers was performed according to Neilan et al. [43]. For the amplification of *mcyE*/*ndaF* gene, the same primers (HEPF and HEPR) and PCR cycling conditions as in Jungblut and Neilan [44] were used. The PCRs were run in 25 μL solution containing approximately 100 ng of DNA, 5 pmol of each specific oligonucleotide primer, 12.5 μL MyTaq™ Red Mix (Bioline Reagents Ltd., London, UK) and 1 μg/μL bovine albumin (Sigma-Aldrich, Saint Louis, MO, USA). MilliQ water was used as a negative control, and the DNA isolated from *N. spumigena* strains CCNP1401 and CCY9414 [12] were used as a positive control. The PCR products were purified with ExtractMe DNA clean-up kit (Blirt S.A., Gdansk, Poland) and sequenced with respective forward and reverse primers (Genomed S.A., Warszawa, Poland). The obtained nucleotide sequences were edited with Chromas Lite 2.1 and deposited in the DDBJ/EMBL/GenBank databases under accession numbers listed in Figure S4. The 16S rDNA sequences obtained from Oslofjorden were aligned using MEGA version 7 [45] and the alignment was corrected manually. Neighbour-joining, maximum likelihood and maximum parsimony phylogenetic trees were constructed with MEGA version 7. For each tree, a bootstrap analysis of 1000 replications was performed.

### 5.4. Sediment ^14^C-Dating

Radiocarbon dating was performed to establish the age of nine selected sediments layers of the cores (Table S2). For this purpose, the lyophilized samples of sediments were treated with 1 M HCl (80 °C) for a few hours until the emanation of gas bubbles was no longer visible. After acid treatment, the samples were rinsed with deionized water until pH = 7. Then, sediment samples were graphitized using an Automated Graphitization Equipment AGE-3 (IonPlus AG) prior to the measurements with a single-stage accelerator mass spectrometer (SSAMS, NEC, USA). The accuracy of the measured ^14^C/^12^C ratio was better than 0.3%. Phthalic acid (Merck) was used for the estimation of the processed background; it was determined to be 2.45 × 10^−3^ *f_M_*. The IAEA-C7 standard was used as reference material. All ^14^C dates were calibrated using the Marine13 dataset (Figure S3) [46] within CALIB 7.1 [47]. Marine reservoir corrections were derived from ^14^C CHRONO Marine Reservoir Database (http://calib.org/marine/, accessed 31 March 2020). Based on the obtained chronology, the age-depth models for the LCs from Oslofjorden and Balsfjorden were generated using polynomial regressions. Because of limited data available (one result), the construction of an age-depth model for the core from Trondheimsfjorden was not possible.

## Figures and Tables

**Table 1:** Quality/quantity of DNA isolated from the short (SC) and long (LC) cores from Norwegian fjords and PCR products of the sequences specific to cyanobacteria and microcystin/nodularin (+ positive result; - negative result; * lyophilized sediments).

Sample	Sediment Layer [cm]	DNA [ng/µL]	A_260/280_	16S rDNA	*cpcBA*-IGS	*mcyE*/*ndaF*
*N. spumigena* CCNP1401				+	+	+
*N. spumigena* CCY9414				+	+	+
Oslofjorden SC	0–1	242	1.2	+	-	-
	1–5	139	1.3	+	-	-
	5–10	140	1.3	+	-	-
	10–15	210	1.3	-	-	-
	15–20	100	1.3	+	-	-
Oslofjorden LC	32–34	72	1.2	+	+	-
	34–36	118	1.4	+	+	+
	42–44	75	1.2	+	-	+
	68–70	44	1.3	-	+	-
	84–86	70	1.2	+	+	+
	88–90	71	1.2	+	+	-
	114–116	85	1.1	+	+	+
	126–128	91	1.2	+	+	+
	128–130	96	1.2	-	+	+
	146–148	140	1.1	-	-	+
	148–150	132	1.2	+	+	+
	178–180	62	1.3	+	+	+
	180–182	105	1.2	+	+	-
	224–226	86	1.2	+	-	-
	232–234	40	1.1	+	+	+
	260–262	19	1.4	+	+	-
	262–264	50	1.2	+	-	+
	314–316	47	1.2	+	+	-
	334–336	46	1.2	+	+	-
	336–338	31	1.2	+	+	-
	338–340	40	1.3	+	+	+
	362–364	31	1.3	+	-	+
	364–366	38	1.8	+	-	-
Balsfjorden LC *	152–154	64	1.2	+	-	-
	220–222	45	1.2	+	-	-
	230–232	177	1.2	+	-	-
Trondheimsfjorden LC *	92–94	65	1.0	+	+	-
	134–136	-	-	-	-	-

**Table 2:** Radiocarbon age (BP) of sediments from the Norwegian fjords and the Baltic Sea where the peak nodularin content (NOD) was recorded.

Presenceof NOD in the Sediment Core	Norwegian Fjords			Baltic Sea
	Balsfjorden	Trondheimsfjorden	Oslofjorden	Gdańsk Depth
NOD Peaks [cal yr BP]			2330–2660	~2500 *
	3540–3830	4200–4520	4360–4690	~4200 *
			7100–7370	
	4820–5140		1100–1130	

* Based on data published by Cegłowska et al. [13] and Szymczak-Żyła et al. [29].

**Table 3:** Basic characteristics of the sampling stations in the Norwegian fjords.

Sampling Station	Depth [m]	Core Length [cm]
Oslofjorden 59° 50.648′ N; 10° 43.560′ E	77	336
Trondheimsfjorden 63° 28.370′ N; 10° 11.650′ E	502	136
Balsfjorden 69° 17.361′ N; 19° 22.586′ E	112	440

## Supplementary Materials

The following are available online at https://www.mdpi.com/2072-6651/12/4/257/s1, Figure S1. MRM chromatogram of nodularin (NOD) extracted from 30–32 cm layer of the long core (LC) collected in Oslofjorden, Figure S2. Enhanced product ion mass spectra (EPI) of nodularin (NOD) extracted from the long sediment core collected in Balsfjorden, Figure S3. Age-depth models for two studied long sediment cores (Oslofjorden and Balsfjorden). The sedimentation rate ranges are given in brackets. The model shows that the sedimentation rates for Trondheimsfjorden were higher (0.53–0.59 mm/yr), but their variations are lower, compared to Oslofjorden (0.20–0.43 mm/yr). In Oslofjorden sedimentation rate in older times was lower (0.20 mm/yr) than in recent times (0.32–0.43 mm/yr), Figure S4. Neighbour-joining phylogenetic tree based on the 16S rDNA sequences (504 bp) obtained from DNA isolated from Oslofjorden (marked in blue) and reference sequences (retrieved from NCBI). Phylogenetic relationships were bootstrapped 1000 times, Table S1. Changes in nodularin contents (NOD) [ng/g dw] and changes in relative amounts of anabaenopeptin AP883a (expressed as a ratio of AP peak area/g dw) in the short core (SC) and long core (LC) from Oslofjorden (* results for individual 2 cm layers), Table S2. Calibrated age of sediment samples from the Norwegian fjords, Table S3. Changes in nodularin contents (NOD) [ng/g dw] and changes in relative amounts of anabaenopeptin AP883a (expressed as a ratio of AP peak area/g dw) in the long core (LC) from Trondheimsfjorden (* results for individual 2 cm layers; # results for integrated 10 cm sections), Table S4. Changes in nodularin contents (NOD) [ng/g dw] and changes in relative amounts of anabaenopeptins (APs) (expressed as a ratio of AP peak area/g dw) in the long core (LC) from Balsfjorden (* results for individual 2 cm layers; # results for integrated 10 cm sections).
