# Supplementary material for: Blooms of Toxic Cyanobacterium Nodularia spumigena in Norwegian Fjords During Holocene Warm Periods
Source: Toxins (Basel). 2020 Apr 15;12(4):257. doi: 10.3390/toxins12040257 (PMC7232221; doi:10.3390/toxins12040257)
Supplement: Supplementary file 1 [file toxins-12-00257-s001.pdf]

Supplementary Materials: Blooms of the toxic cyanobacterium *Nodularia spumigena* in Norwegian fjords during Holocene warm periods

Robert Konkel, Anna Toruńska-Sitarz, Marta Cegłowska, Žilvinas Ežerinskis, Justina Šapolaitė, Jonas Mažeika and Hanna Mazur-Marzec

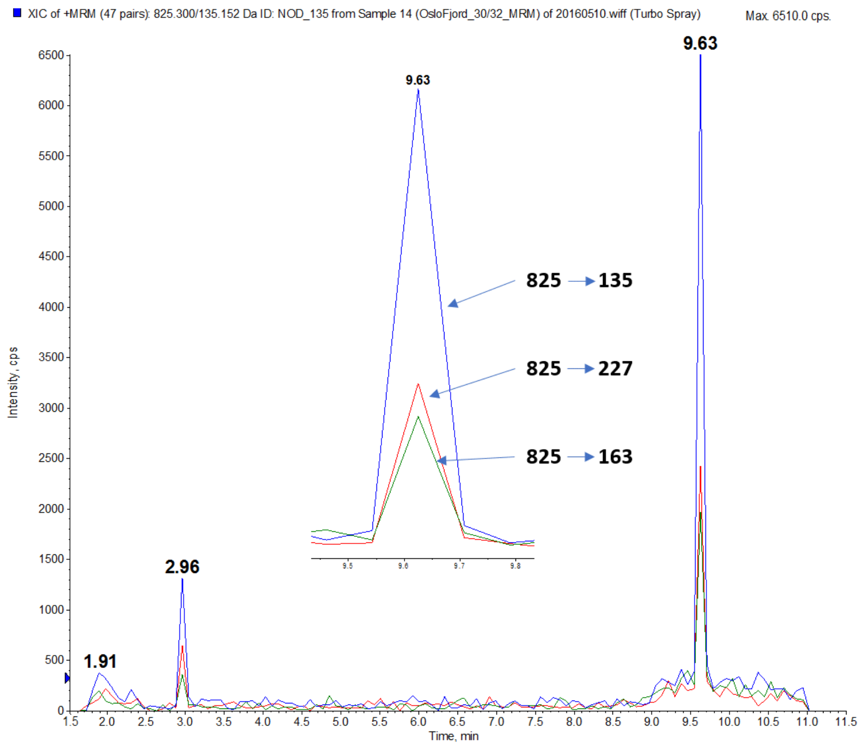

Figure S1. MRM chromatogram of nodularin (NOD) extracted from 30–32 cm layer of the long core (LC) collected in Oslofjorden.

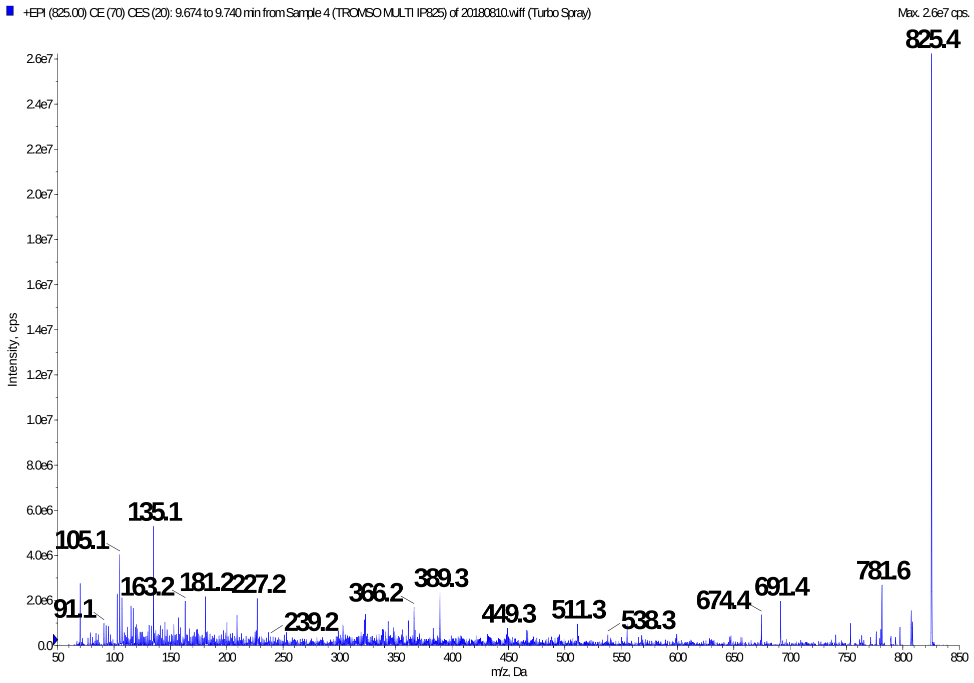

Figure S2. Enhanced product ion mass spectra (EPI) of nodularin (NOD) extracted from the long sediment core collected in Balsfjorden.

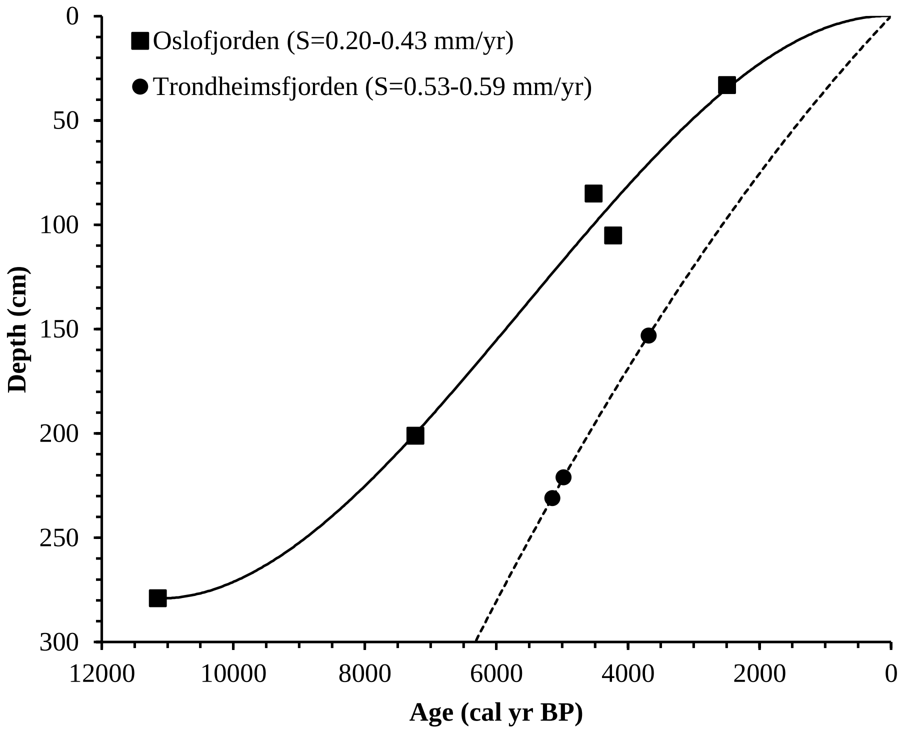

Figure S3. Age-depth models for two studied long sediment cores (Oslofjorden and Balsfjorden). The sedimentation rate ranges are given in brackets. The model shows that the sedimentation rates for Trondheimsfjorden were higher (0.53–0.59 mm/yr), but their variations are lower, compared to Oslofjorden (0.20–0.43 mm/yr). In Oslofjorden sedimentation rate in older times was lower (0.20 mm/yr) than in recent times (0.32–0.43 mm/yr).

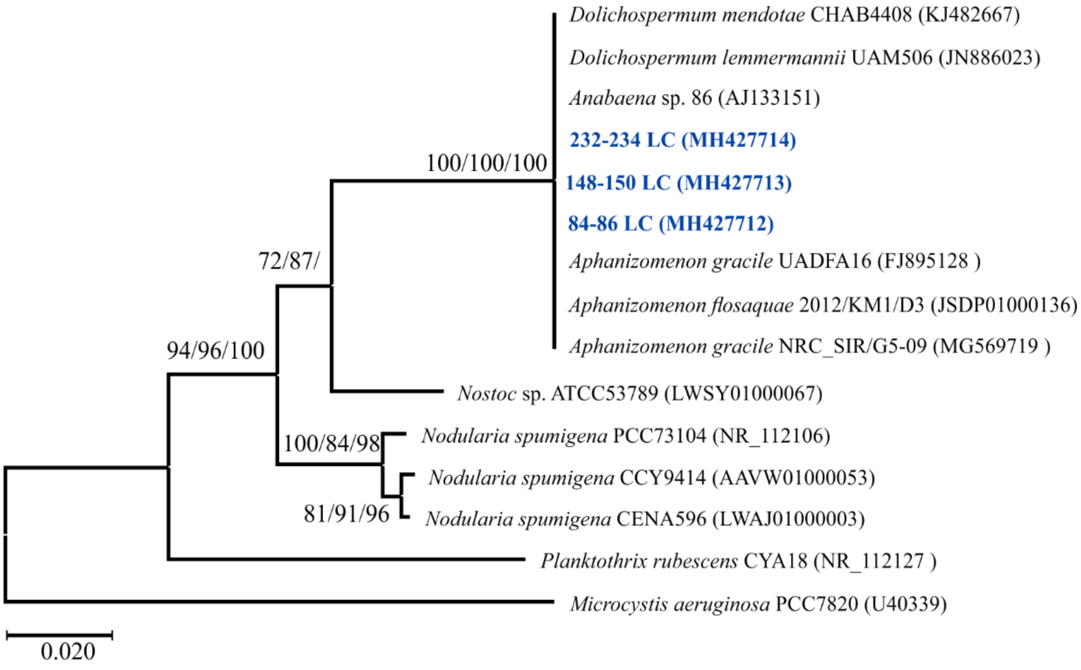

Figure S4. Neighbour-joining phylogenetic tree based on the 16S rDNA sequences (504 bp) obtained from DNA isolated from Oslofjorden (marked in blue) and reference sequences (retrieved from NCBI). Phylogenetic relationships were bootstrapped 1000 times.

Table S1. Changes in nodularin contents (NOD) [ng/g dw] and changes in relative amounts of anabaenopeptin AP883a (expressed as a ratio of AP peak area/g dw) in the short core (SC) and long core (LC) from Oslofjorden (* results for individual 2 cm layers).

Core layer [cm]	NOD [ng/g dw]	AP 883a peak area
**Short core (SC)**		
**0–8***	-	-
**8–10**	1.15	597.49
**10–40***	-	-
**Long core (LC)**		
**0–2**	1.21	-
**2–4**	0.32	traces
**4–6**	0.47	1530.13
**6–8**	0.85	1135.28
**8–10**	0.90	1727.59
**10–12**	1.09	691.04
**12–14**	1.64	-
**14–16**	1.26	-
**16–18**	1.56	traces
**18–20**	0.98	traces
**20–22**	1.54	1382.06
**22–24**	1.49	-
**24–26**	1.16	789.76
**26–28**	1.25	1283.33
**28–30**	1.33	1135.24
**30–32**	1.80	937.83
**32–34**	3.84	1283.36
**34–36**	1.27	-
**36–38**	1.15	2023.72
**38–40**	1.76	-
**40–42**	1.17	-
**42–43**	1.53	1233.99
**44–46**	0.52	1085.92
**46–48**	0.69	-
**48–50**	0.62	-
**50–52**	0.74	2369.23
**52–54**	0.84	740.40
**54–56**	0.95	-
**56–58**	0.94	1678.23
**58–60**	1.31	-
**60–62**	1.04	-
**62–64**	1.09	-
**64–66**	1.09	traces
**66–68**	0.99	-
**68–70**	1.14	-
**70–72**	1.22	-
**72–74**	1.02	-
**74–76**	1.35	-
**76–78**	1.02	traces
**78–80**	1.11	-
**80–82**	1.08	937.81
**82–84**	1.11	-
**84–86**	1.68	-
**86–88**	1.53	-
**88–90**	1.21	-
**90–92**	1.21	-
**92–94**	0.67	-

**94–96**	0.43	-
**96–98**	0.84	-
**98–100**	1.57	-
**100–102**	0.80	-
**102–104**	0.37	-
**104–106**	0.33	-
**106–108**	0.27	-
**108–110**	0.52	-
**110–112**	0.65	-
**112–114**	0.61	-
**114–116**	0.82	-
**116–118**	0.29	-
**118–120**	0.82	-
**120–122**	0.63	-
**122–124**	0.50	-
**124–126**	0.35	-
**126–128**	0.39	-
**128–130**	0.33	-
**130–132**	0.31	-
**132–134**	0.52	-
**134–136**	0.50	-
**136–138**	0.45	-
**138–140**	0.58	-
**140–142**	0.28	-
**142–144**	0.32	-
**144–146**	0.31	-
**146–148**	0.28	-
**148–150**	0.21	-

Core layer [cm]	NOD [ng/g dw]	AP 883a peak area
**Long core (LC)**		
**152–154**	0.23	-
**154–156**	0.32	-
**156–158**	0.29	-
**158–160**	0.30	-
**160–162**	0.29	-
**162–164**	0.35	-
**164–166**	0.24	-
**166–168**	0.23	-
**168–170**	0.26	-
**170–172**	0.21	-
**172–174**	0.37	-
**174–176**	0.31	-
**176–178**	0.29	-
**178–180**	0.44	-
**180–182**	0.33	-
**182–184**	0.30	-
**184–186**	0.46	-
**186–188**	0.31	-
**188–190**	0.34	-
**190–192**	0.36	-
**192–194**	0.48	-
**194–196**	0.14	-
**196–198**	0.45	-
**198–200**	0.66	-
**200–202**	0.45	-
**202–204**	0.49	-
**204–206**	0.43	-

**206–208**	0.48	-
**208–210**	0.53	-
**210–212**	0.48	-
**212–214**	0.07	-
**214–216**	0.07	-
**216–218**	-	-
**218–220**	0.08	-
**220–222**	-	-
**222–224**	0.07	-
**224–226**	0.07	-
**226–228**	-	-
**228–230**	-	-
**230–232**	-	-
**232–234**	-	-
**234–236**	-	-
**236–238**	0.03	-
**238–240**	0.14	-
**240–242**	0.19	-
**242–244**	0.23	-
**244–246**	0.23	-
**246–248**	0.29	-
**248–250**	0.18	-
**250–252**	0.21	-
**252–254**	0.21	-
**254–256**	0.34	-
**256–258**	0.20	-
**258–260**	0.26	-
**260–262**	0.22	-
**262–264**	0.08	-
**264–266**	0.15	-
**266–268**	0.13	-
**268–270**	0.07	-
**270–272**	0.10	-
**272–274**	0.06	-
**274–276**	-	-
**276–278**	0.19	-
**278–280**	0.10	-
**280–282**	0.08	-
**282–284**	-	-
**284–286**	0.30	-
**286–288**	-	-
**288–290**	-	-
**290–292**	-	-
**292–294**	0.53	-
**294–296**	0.08	-
**296–298**	-	-
**298–300**	0.11	-
**300–302**	-	-
**302–304**	0.06	-
**304–334***	-	-

Table S2. Calibrated age of sediment samples from the Norwegian fjords.

Sampling station Layer [cm]	Calibrated age [yr BP]
Balsfjorden 152–154	3540–3830
Balsfjorden 220–222	4820–5140
Balsfjorden 230–232	4990–5310
Oslofjorden 32–34	2330–2660
Oslofjorden 84–86	4360–4690
Oslofjorden 104–106	4070–4380
Oslofjorden 200–202	7100–7370
Oslofjorden 278–280	11000–11300
Trondheimsfjorden 92–94	4200–4520

Table S3. Changes in nodularin contents (NOD) [ng/g dw] and changes in relative amounts of anabaenopeptin AP883a (expressed as a ratio of AP peak area/g dw) in the long core (LC) from Trondheimsfjorden (*results for individual 2 cm layers; # results for integrated 10 cm sections).

Core layer [cm]	NOD [ng/g dw]	AP	Calibrated age[yr BP]
**Long core (LC) (integrated 10-cm sections)**			
0–60#	-	-	
60–70	0.05	-	
70–80	0.02	-	
80–90	0.11	-	
90–100	0.63	AP 883a	
100–110	0.04	-	
110–120	0.08	-	
120–130	0.03	-	
130–136	0.07	-	
**Long core (LC) (2-cm layers)**			
0–90*	-	-	
90–92	0.09	AP 883a	
92–94	0.27	AP 883a	4200–4520
94–136*	-	-	

Table S4. Changes in nodularin contents (NOD) [ng/g dw] and changes in relative amounts of anabaenopeptins (APs) (expressed as a ratio of AP peak area/g dw) in the long core (LC) from Balsfjorden (*results for individual 2 cm layers; # results for integrated 10 cm sections).

Core layer [cm]	NOD [ng/g dw]	AP	Calibrated age [yr BP]
**Long core (LC) (integrated 10 cm sections)**			
0–140#	-	-	
140–150	0.14	-	
150–160	0.22	AP 827	
160–220#	-	-	
220–230	0.80	AP 883a	
230–240	1.19	-	
240–260#	-	-	
260–270	0.18	-	
270–440#	-	-	
**Long core (LC) (2-cm layers)**			
0–152*	-	-	
152–154	traces	-	3540–3830
154–204*	-	-	
204–206	0.10	-	
206–220*	-	-	
220–222	0.58	AP 883a	4820–5140
222–224	0.08	-	
224–226	0.05	-	
226–228*	-	-	
228–230	0.02	-	
230–232	0.41	-	4990–5310
232–234	-	-	
234–236	0.40	-	
236–238	0.19	-	
238–240	0.10	-	
262–272*	-	-	
